# (Dys)regulation of Synaptic Activity and Neurotransmitter Release by β-Amyloid: A Look Beyond Alzheimer's Disease Pathogenesis

**DOI:** 10.3389/fnmol.2021.635880

**Published:** 2021-02-24

**Authors:** Francesca Fagiani, Cristina Lanni, Marco Racchi, Stefano Govoni

**Affiliations:** ^1^Department of Drug Sciences, Pharmacology Section, University of Pavia, Pavia, Italy; ^2^Istituto Universitario di Studi Superiori – IUSS, Pavia, Italy

**Keywords:** β-amyloid, Alzheimer's disease, synaptic activity, neurotransmission, neuropsychiatric symptoms, synaptic vesicle cycle

## Abstract

Beside its widely studied role in the pathogenesis of Alzheimer's disease (AD), β-amyloid (Aβ) is a normal and soluble product of neuronal metabolism that regulates several key physiological functions, exerting neuromodulatory effects on synaptic plasticity, memory, and neurotransmitter release. Such effects have been observed to occur in a hormetic fashion, with Aβ exhibiting a dual role influenced by its concentration, the different isoforms, or aggregation forms of the peptide. However, to date, our knowledge about the physiological functions of Aβ and, in particular, its modulatory role on synaptic activity and neurotransmission in the normal brain is fragmentary, thus hindering a clear comprehension of the biological mechanisms underlying the derangement from function to dysfunction. In particular, according to the amyloid cascade hypothesis, the switch from physiology to pathology is linked to the abnormal increase in Aβ levels, due to an imbalance in Aβ production and clearance. In this regard, increased Aβ levels have been hypothesized to induce early defects in synaptic function and such alterations have been suggested to account, at least in part, for the onset of neuropsychiatric symptoms (e.g., apathy, anxiety, changes in mood, depression, and agitation/aggression), frequently observed in the prodromal stage of AD. Therefore, understanding the biological mechanisms underlying early synaptic alterations in AD is a key starting point to frame the relevant time windows for AD treatment and to gain insight into AD etiopathogenesis.

## Introduction

β-amyloid (Aβ) is a 4-kDa peptide produced during the amyloidogenic pathway by the sequential proteolysis of the amyloid precursor protein (APP) by β- and γ-secretase (Glenner and Wong, 1984). APP is first cleaved by β-site APP cleaving enzyme one (BACE1), thereby releasing the C-terminal fragment (C99), further cleaved by γ-secretase to generate Aβ peptide. Aβ is secreted into the extracellular space and cleared by the cerebrospinal fluid (CSF) and vascular system (Iliff et al., 2012). In the CSF of healthy humans, the 40-amino-acid-long isoform (Aβ_1−40_) has been reported to be the most represented isoform, which is present at ~2–3 ng/ml, and the 42-amino-acid-long isoform (Aβ_1−42_) to be the second most abundant isoform (~0.75 ng/ml) (Ida et al., 1996; Mo et al., 2015). Aβ is normally present in a soluble form, but it can self-assemble. In particular, Aβ_1−42_ has been reported to be more prone to aggregation than Aβ_1−40_, which differ from the former by two amino acid residues at the C-terminal end (Sgourakis et al., 2007). The C-terminal flexibility of the Aβ_1−42_ peptide has been suggested to be responsible for its higher propensity to aggregate (Sgourakis et al., 2007). The self-assembly of Aβ produces aggregates such as oligomers that can further accrete to form protofibrils, fibrils, and, finally, insoluble plaques, one of the main histological hallmarks observed in the brain of Alzheimer's disease (AD) patients.

The pathological role of Aβ as a misfolded protein involved in the pathogenesis of AD, according to the amyloid hypothesis (Hardy and Selkoe, 2002), has been extensively investigated for decades, with different types of Aβ oligomers found to correlate with cognitive impairment and to promote neurodegeneration in AD (Walsh and Selkoe, 2007; Ono et al., 2009). Because of this evidence reporting Aβ key role in several physiological functions (for a comprehensive review on the topic see Brothers et al., 2018) has been partially overshadowed. Since Aβ is released into the extracellular space as monomer, the physiological roles of Aβ have been commonly ascribed to its monomeric form (Giuffrida et al., 2009). However, the dynamic equilibrium between monomers/oligomers in the brain under physiological conditions is still matter of scientific debate (Bemporad and Chiti, 2012). It has been suggested that a certain degree of Aβ oligomerization may also occur under physiological conditions (Gulisano et al., 2018). In this regard, although the effects of different forms and aggregation status of soluble Aβ have been widely investigated for their well-established neurotoxic potential in AD, less is known about such aspects under physiological conditions. In the following sections, we will discuss evidence from the literature reporting the effects of low (i.e., picomolar–nanomolar) concentrations of the main Aβ isoforms (i.e., Aβ_1−40_ and Aβ_1−42_), as well as of different aggregation status of Aβ (i.e., monomers, low-weight-soluble oligomers, and combination of both) at synaptic level, in condition not resulting in neurotoxicity.

## The Neuromodulatory Role of Aβ

Among the physiological functions regulated by Aβ, several lines of evidence indicate that Aβ exerts a neuromodulatory role by controlling synaptic activity and neurotransmitter release from presynaptic terminals (Preda et al., 2008; Puzzo et al., 2008, 2011; Abramov et al., 2009; Grilli et al., 2010; Mura et al., 2012; Zappettini et al., 2012). Aβ has been demonstrated to act in a hormetic fashion, exhibiting a dual role on synaptic activity and neurotransmission, strictly depending upon its concentration. Accordingly, while low Aβ concentrations (picomolar and low nanomolar), resembling the endogenous levels of Aβ in the brain, have been found to positively modulate neurotransmission and memory, higher concentrations (high nanomolar–low micromolar) have been observed to negatively modulate neurotransmission, finally resulting in the well-established neurotoxic action (Puzzo et al., 2008). Moreover, these opposite Aβ effects have been demonstrated to be influenced not only by Aβ concentration but also by different isoforms or aggregation forms of the peptide (Gulisano et al., 2018).

In addition, a number of studies demonstrates that, at presynaptic terminals, Aβ regulates the release of neurotransmitters, including dopamine, γ-aminobutyric acid (GABA), glutamate, aspartate, and glycine, by mainly affecting the cholinergic control of their release, in conditions not resulting in neurotoxicity (Preda et al., 2008; Grilli et al., 2010; Mura et al., 2012; Zappettini et al., 2012). In accordance with such evidence, Aβ has been found to act as positive endogenous modulator of release probability at hippocampal synapses (Abramov et al., 2009), and a direct and indirect interplay of Aβ with different presynaptic proteins regulating the sequential steps (i.e., exocytosis, endocytosis, and trafficking) of synaptic vesicle cycle at presynaptic terminals has been reported (Russell et al., 2012; Yang et al., 2015; Lazarevic et al., 2017). However, data concerning the physiological functions of Aβ and, in particular, the modulation of synaptic activity and neurotransmission by Aβ in the normal brain are still fragmentary, thus hindering a clear comprehension of the biological mechanisms underlying the derangement from function to dysfunction. According to the amyloid cascade hypothesis, the switch from physiology to pathology is linked to the abnormal increase in Aβ levels, due to an imbalance in Aβ production and clearance (Mawuenyega et al., 2010; Murphy and Levine, 2010). In this regard, increased Aβ levels have been hypothesized to induce early defects in synaptic activity and neurotransmission, and such alterations have been suggested to account, at least in part, for the onset of early behavioral symptoms, including apathy, anxiety, changes in mood, depression, and psychosis, frequently observed in the prodromal stage of AD (reviewed by Ismail et al., 2016). Therefore, such early defects may likely be the consequences of synaptic dysfunction rather than of neurodegenerative processes. Understanding the biological mechanisms underlying early synaptic alterations in AD might represent a key starting point to better frame the relevant time windows and to gain insight into AD etiopathogenesis, as well as defining the associated early behavioral signs.

### Aβ as Endogenous Regulator of Synaptic Activity

Independent *in vitro* and *in vivo* studies have demonstrated that neuronal activity directly increases Aβ production and secretion into the extracellular space at the synapses (Kamenetz et al., 2003; Cirrito et al., 2005) and that, in turn, Aβ suppresses excitatory synaptic transmission, thereby maintaining neuronal activity within a normal dynamic range (Kamenetz et al., 2003). In this regard, it has been speculated that such negative feedback loop may act as a physiological homeostatic mechanism to limit the overexcitation of brain circuits that might result in excitotoxicity (Kamenetz et al., 2003). Thus, deviation from this fine-tuning control mechanism due to Aβ derangement may suppress synaptic activity and, ultimately, lead to synaptic damage. However, the concentrations of Aβ tested in these studies are far higher than the endogenous levels of Aβ peptides in the normal brain, estimated in the picomolar range (Seubert et al., 1992; Ida et al., 1996), and prompted investigations on the effects of low Aβ concentrations (i.e., picomolar and low nanomolar) resembling its endogenous levels. In this regard, a growing body of evidence converges to indicate that soluble Aβ acts as a crucial synaptic regulator, by modulating key physiological functions, such as synaptic plasticity and memory. Accordingly, Puzzo et al. (2008) demonstrated that the exposure of hippocampal neurons to low concentrations (i.e., picomolar–low nanomolar) of Aβ_1−42_ positively modulated synaptic plasticity and memory. In contrast, the exposure to higher concentrations (i.e., high nanomolar–low micromolar) induced a neurotoxic action. In particular, Aβ_1−42_ exhibited a biphasic or hormetic effect in regulating the long-term potentiation (LTP), the electrophysiological correlate of learning, and memory (Puzzo et al., 2008). Accordingly, at the synapses between Schaffer collateral fibers and CA1 neurons, picomolar concentrations of Aβ_1−42_ promoted LTP enhancement, with a maximum effect around 200 pM, whereas nanomolar concentrations of Aβ_1−42_ induced an impairment of LTP. Moreover, picomolar concentrations of Aβ_1−42_ induced an enhancement of both hippocampal-dependent reference and contextual fear memory in mice. In line with these data, in mouse hippocampal slices, perfusion with the monoclonal antibody JRF/rAb2, recognizing a specific epitope of rodent Aβ_1−40_ and Aβ_1−42_, led to a decrease in contextual fear memory and reference memory, as well as significantly reduced LTP (Puzzo et al., 2011). Both these parameters were rescued by the addition of the human homolog Aβ_1−42_, which is not recognized by JRF/rAb2, suggesting that endogenous Aβ might be required for synaptic plasticity in the brain. According to this hypothesis, intraneuronal delivery of a small interfering RNA (siRNA), specific for rodent APP, induced a reduction in LTP that was rescued by the addition of 200 pM Aβ_1−42_.

Furthermore, the effects of soluble Aβ on synaptic plasticity and memory have been reported to rely not only on the concentration of Aβ but also on the different isoforms and aggregation status of the peptide. Accordingly, Gulisano et al. (2018) demonstrated that the exposure of rodent CA1 pyramidal neurons to 200 pM low-molecular-weight oligomeric Aβ_1−42_ led to an increase of frequency in miniature excitatory postsynaptic currents, accompanied by a reduction in pair pulse facilitation. In addition, an increased number of docked vesicles at presynaptic terminals was also observed, thus suggesting that low concentrations of oligomeric Aβ_1−42_ promote neurotransmitter release from the presynaptic terminals. Noteworthy, such effects were not observed when pyramidal neurons were exposed to 200 pM Aβ_1−40_ oligomers. Moreover, although monomeric forms of Aβ are commonly considered as neuroprotective (Giuffrida et al., 2009), the exposure of neurons to high concentrations (200 nM) of Aβ_1−42_ monomers induced an impairment in synaptic plasticity and memory (Gulisano et al., 2018). By contrast, such effect was not observed for Aβ_1−40_ monomers. Indeed, the exposure to 200 nM Aβ_1−40_ monomers was ineffective, whereas 200 nM Aβ_1−40_ oligomers impaired synaptic plasticity and memory. However, while interpreting these results, it should be taken into account that the preparation of Aβ_1−42_ monomers, which promoted the observed neurotoxic action, contained also few dimers and higher quantity of trimers and tetramers that may be responsible for neurotoxicity.

Moreover, also the time of exposure to the peptide represents a key parameter to consider. In this regard, Koppensteiner et al. (2016) observed that, in mouse hippocampal neurons, short-term exposure (minutes) to picomolar concentration (200 pM) of oligomeric Aβ_1−42_ stimulated synaptic potentiation in hippocampal cultures and slices and synaptic plasticity and contextual memory in mice. Differently, longer exposures (hours) to 200 pM Aβ_1−42_ induced a decrease in such parameters. In this regard, it is important to consider that dynamic Aβ changes physiologically occur in the brain, since Aβ levels undergo diurnal fluctuations. Accordingly, both in mouse hippocampal interstitial fluid as well as in human CSF, soluble Aβ levels have been reported to exhibit robust daily oscillations, with a clear 24-h period, that are in phase with circadian rhythms in activity (Kang et al., 2009; Huang et al., 2012), thus indicating the presence of physiological circadian patterns regulating fluctuations of CSF Aβ levels. Notably, Huang et al. (2012) demonstrated that aging and Aβ deposition diminish normal CSF Aβ dynamics to a flat line, possibly contributing to AD.

### Aβ as Endogenous Regulator of Neurotransmitter Release

Evidence from the literature indicates that Aβ controls neurotransmitter release from presynaptic terminals in the absence of evident signs of neurotoxicity. A functional interplay between Aβ and different neurotransmitter systems, such as cholinergic, glutamatergic, GABAergic, catecholaminergic, and serotoninergic, has been reported (for a comprehensive review on the topic, see Lanni et al., 2019). It has been speculated that Aβ exhibits a neuromodulatory action fundamental for the proper balance among the different neurotransmitter systems.

Notably, Aβ has been found to regulate the cholinergic control of neurotransmitter release in several brain regions in a concentration-dependent manner in different *in vitro* and *in vivo* models, as schematized in detail in Table 1. Both Aβ_1−40_ and Aβ_1−42_ isoforms have been demonstrated to bind with high affinity to α7-containing nicotinic acetylcholine receptors (α7-nAChRs) (Wang et al., 2000; Khan et al., 2010; Tong et al., 2011). Picomolar–low nanomolar concentrations of Aβ_1−40_ have been found to activate α7-nAChRs, thus triggering intracellular pathways regulating synaptic plasticity, learning, and memory. Conversely, higher concentrations (nanomolar–low micromolar), as well as prolonged exposure to Aβ_1−40_, have been found to desensitize and inactivate α7-nAChRs, thereby disrupting synaptic signaling (Mura et al., 2012; Zappettini et al., 2012). Taken together, these results converge to indicate that, while Aβ may physiologically exert a neuromodulatory action on nicotinic receptors, its accumulation, whose primary etiological factors may be an imbalance between Aβ production and its clearance (Mawuenyega et al., 2010; Murphy and Levine, 2010), may damage nicotinic transmission, by inducing the inactivation of α7-nAChRs, with consequent impairment of nicotinic cholinergic neurotransmission.

**Table 1:** Regulation by Aβ of cholinergic control of neurotransmitter release.

**Aβ species**	**Concentration and timing**	**Effects of Aβ on neurotransmitter release**	**Experimental model/brain area**	**References**
**Dopamine**				
Soluble Aβ_1−40_ and Aβ_1−42_	1–10 μM/60–80 min (for *in vivo* experiments); 100 nM/up to 10 min (for *in vitro* experiments)	Low micromolar concentrations (1 μM) of Aβ prevented the muscarinic receptor-activated dopamine release in rat nucleus accumbens. The [^3^H]dopamine release, evoked by carbachol, was decreased by 100 nM Aβ in isolated nerve endings of the nucleus accumbens. Moreover, Aβ_1−42_ (100 nM) significantly reduced the dopamine release evoked by carbachol.	*In vivo* (brain dialysis) and *in vitro* (isolated synaptosomes) models/rat nucleus accumbens	Preda et al., 2008
Aβ_1−40_	100 nM	Treatment with 100 nM Aβ_1−40_ prevented both nicotinic and muscarinic cholinergic modulation of dopamine release.	Synaptosomes/rat nucleus accumbens	Olivero et al., 2014
Aβ_1−40_ and Aβ_1−42_	10–100 nM/up to 12 min	In nerve endings, Aβ impaired the muscarinic control of dopamine release in both the nucleus accumbens and caudate putamen.	Synaptosomes/caudate-putamen-nucleus accumbens	Mura et al., 2010
**GABA**				
Monomers of Aβ_1−40_ and Aβ_1−42_	100 nM/up to 17 min	In isolated nerve endings, Aβ blocked GABA release by acting on muscarinic receptor subtypes (M3 and M5). Instead, Aβ was ineffective on muscarinic receptor subtypes negatively modulating the stimulated transmitter release (M2 and M4).	Synaptosomes/rat nucleus accumbens	Grilli et al., 2010
Monomers of Aβ_1−40_	100 nM, 1 μM, and 10 μM/40–60 min (for *in vivo* experiments); 100 pM, 1 nM, and 100 nM/up to 10 min (for *in vitro* experiments)	While perfusion of 10 μM Aβ blocked the nicotine-induced release of GABA, perfusion of 100 nM Aβ potentiated the nicotine-evoked GABA overflow. In isolated nerve endings, 100 nM Aβ blocked the nicotine-induced release of GABA and 100 nM Aβ inhibited the release of GABA induced by the 4β2 selective agonist 5IA85380.	*In vivo* (microdialysis) and *in vitro* (synaptosomes in superfusion) techniques/hippocampus	Mura et al., 2012
**Glycine**				
Aβ_1−40_	10 μM/40–60 min (for *in vivo* experiments); 10 nM and 100 nM/up to 10 min (for *in vitro* experiments)	Perfusion of 10 μM Aβ_1−40_ reduced the nicotine-induced glycine overflow and also the glycine overflow induced by the α7 selective agonist PHA543613. In isolated nerve endings, both 10 and 100 nM Aβ inhibited the nicotine-induced glycine release; 100 nM Aβ inhibited the release of glycine evoked by the α7 selective agonist carbachol and by the α4β2 selective agonist 5IA85380.	*In vitro* (synaptosomes in superfusion) and *in vivo* (microdialysis) approaches/hippocampus	Zappettini et al., 2012
**Aspartate**				
Monomers of Aβ_1−40_	100 nM, 1 μM, and 10 μM/40–60 min (for *in vivo* experiments); 100 pM, 1 nM, and 100 nM/up to 10 min (for *in vitro* experiments)	Perfusion of 10 and 1 μM Aβ inhibited the nicotine-induced release of aspartate. In isolated nerve endings, 100 nM Aβ inhibited the nicotine-induced release of aspartate; 100 nM Aβ inhibited the release of aspartate that was induced by the α7 selective agonist carbachol; 100 nM Aβ inhibited the release of aspartate induced by the α4β2 selective agonist 5IA85380; 100 pM Aβ potentiated the carbachol-induced release of aspartate.	*In vivo* (microdialysis) and *in vitro* (synaptosomes in superfusion) techniques/hippocampus	Mura et al., 2012
**Glutamate**				
Monomers of Aβ_1−40_	100 nM, 1 μM, and 10 μM/40–60 min (for *in vivo* experiments); 100 pM, 1 nM, and 100 nM/up to 10 min (for *in vitro* experiments)	Perfusion of 10 and 1 μM Aβ inhibited the nicotine-induced release of glutamate. In isolated nerve endings, 100 nM Aβ inhibited the nicotine-induced release of glutamate and the release of glutamate induced by the α7 selective agonist carbachol. Instead, 1 nM Aβ potentiated the release of glutamate induced by carbachol; 100 nM Aβ inhibited the release of glutamate induced by the α4β2 selective agonist 5IA85380; 100 pM Aβ potentiated the carbachol-induced release of glutamate.	*In vivo* (microdialysis) and *in vitro* (synaptosomes in superfusion) techniques/hippocampus	Mura et al., 2012

Besides the interaction with cholinergic receptors, low concentrations (range pM–nM) of Aβ_1−40_ also promoted the nicotine-evoked release of both excitatory (i.e., glutamate and aspartate) and inhibitory amino acids (i.e., glycine and GABA) (Mura et al., 2012; Zappettini et al., 2012), while higher concentrations of Aβ_*1–40*_ (range nM–μM) inhibited the nicotine-elicited release of glutamate and aspartate (Mura et al., 2012; Zappettini et al., 2012). These effects are consistent with results obtained in the nucleus accumbens and in the striatum, in the case of GABA and dopamine release upon muscarinic cholinergic stimuli (Preda et al., 2008; Grilli et al., 2010).

#### The Potential Interplay Between Aβ and Synaptic Vesicle Cycle

In an elegant work by Abramov et al. (2009), endogenous Aβ has been demonstrated to exert a pivotal role in the regulation of synaptic vesicle release but not to affect postsynaptic function. In particular, the increase in endogenous Aβ levels, due to the inhibition of its extracellular degradation, led to enhancement of release probability of synaptic vesicles, as well as of neuronal activity in rodent hippocampal culture (Abramov et al., 2009). Such effects increased spontaneous excitatory postsynaptic currents, but not inhibitory currents, and were specifically presynaptic and dependent on firing rates, with lower facilitation observed in hippocampal neurons showing higher firing rates. In line with such evidence reporting Aβ involvement in release probability of synaptic vesicles, evidence from literature indicates that Aβ may directly interact with key presynaptic proteins regulating the neurotransmitter release machinery, by influencing the phosphorylation of SNARE and accessory proteins and, consequently, the assembly of the soluble N-ethylmaleimide-sensitive factor attachment protein (SNAP) receptors (SNARE) complex and the consequent release of neurotransmitter from the presynaptic terminal (Russell et al., 2012; Yang et al., 2015; Marsh et al., 2017). Indeed, Aβ has been reported to interfere with different steps of the synaptic vesicle cycle, such as vesicle docking and fusion, fundamental for the exocytosis of synaptic vesicles, as well as vesicle recycling and recovery in neurons (as reviewed by Fagiani et al., 2019). Corroborating the hypothesis of Aβ implication in the exocytosis of synaptic vesicles, Russell et al. (2012) demonstrated that, at presynaptic terminals, in rat CA3–CA1 hippocampal neurons, monomeric Aβ_1−42_, at nanomolar concentration (50 nM), directly competed with Synaptobrevin/vesicle-associated membrane protein (VAMP2) for the binding to Synaptophysin, thereby promoting the formation of the fusion pore complex, with consequent positive effect on neurotransmitter release. Moreover, Yang et al. (2015) reported that, in an *in vitro* assay, Aβ oligomers (1–20 nM) bind to the SNARE motif region (SynH3) of Syntaxin 1a, thereby inhibiting the fusion step between docking and lipid mixing. Finally, the exposure of rat hippocampal neurons to soluble Aβ oligomers (300 nM) induced an increase in phosphorylated Synapsin I by activating CaMKIV, thereby increasing the availability of synaptic vesicles to dock to the active zone and to promote neurotransmitter release (Marsh et al., 2017). However, these data are extremely limited and do not allow to draw definitive conclusions regarding the functional impact of Aβ on synaptic vesicle cycle. Given the key role of Aβ at presynaptic terminals as well as its effects on neurotransmitter release, discussed above, further studies investigating the interplay between Aβ and the presynaptic release machinery may provide relevant information. In particular, a comparative analysis of the effects of low and high Aβ concentrations, as well as the impact of different soluble species (i.e., monomers and low-weight oligomers) of Aβ peptides, may open new avenues in the field. In this regard, it has to be mentioned that, besides a direct interplay of Aβ with key presynaptic proteins mediating synaptic vesicle dynamics, Aβ has been reported to regulate protein kinases (e.g., calpain-cyclin-dependent kinase 5 and Ca^2+^/calmodulin-dependent protein kinase IV) (Lazarevic et al., 2017; Park et al., 2017), thereby influencing the fine-tuning of synaptic vesicle dynamics at the presynaptic terminal (for a comprehensive review on the topic, see Fagiani et al., 2019).

## Aβ-Driven Dysregulation of Neurotransmission: An Early Event Triggering Behavioral Symptoms in AD?

**Figure 1:**
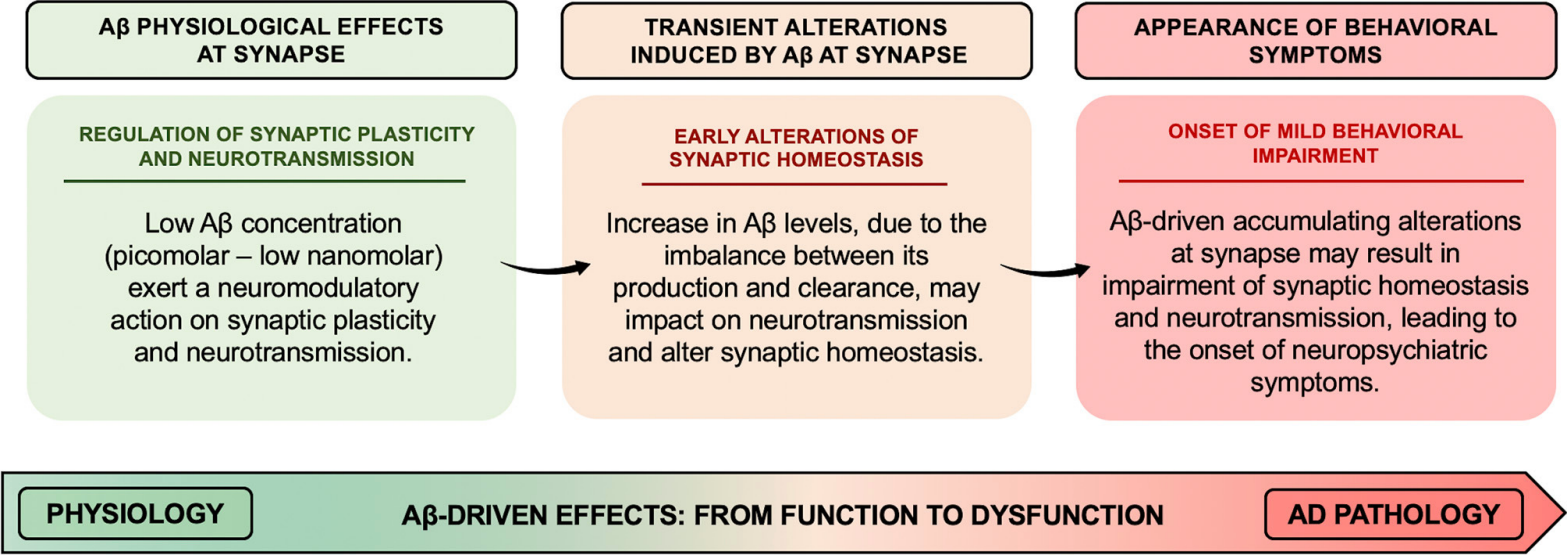
Aβ-driven effects at synapse: the derangement from function to dysfunction. Low concentrations (picomolar–low nanomolar) of Aβ exert a neuromodulatory action on synaptic plasticity and neurotransmitter release from the presynaptic terminals. The alteration of Aβ homeostasis, due to an imbalance between Aβ production and clearance, may impact on the fine-tuning of synaptic vesicle cycling and neurotransmitter release, thus altering synaptic homeostasis. Aβ-driven accumulating alterations at synapse might result in permanent impairment of synaptic homeostasis and neurotransmission, thereby leading to the onset of neuropsychiatric symptoms frequently observed in the prodromal stage of AD.

Pathological increase in Aβ levels has been suggested to lead to the derangement of Aβ neuromodulatory action. In particular, the overall scenario depicted above suggests that increased Aβ levels might transiently affect the fine-tuning of synaptic vesicle cycling and neurotransmitter release, thereby altering synaptic homeostasis, whose accumulating transient alterations may result in long-lasting and even permanent alteration (as illustrated in Figure 1). Therefore, Aβ-induced early synaptic changes altering synaptic homeostasis may promote a linear progression from synaptic dysfunction to frank neurodegeneration (Fagiani et al., 2019). Noteworthy, perturbation of synaptic homeostasis and neurotransmission has been suggested to possibly contribute to the onset of neuropsychiatric symptoms (NPS) (e.g., apathy, social withdrawal, anxiety, changes in mood, depression, agitation/aggression, psychosis, and delusions), frequently observed in the prodromal stage of AD (Ismail et al., 2016). In AD, such behavioral signs have been suggested to be predictive of incipient cognitive decline and to be correlated to early synaptic dysfunction rather than to neurodegenerative processes. In this regard, when thinking of neuropsychiatric manifestations, the first observation is how Aβ-related changes in neurotransmitter release may support and translate, over the time, into the onset of behavioral symptoms (e.g., apathy, anxiety, and depression) (Ismail et al., 2016). For instance, the onset of apathy, one of the main behavioral correlates of the impairment in dopaminergic neurotransmission observed in aging, may be, at least in part, related to the inhibitory effect induced by Aβ on dopamine release (Preda et al., 2008). Furthermore, based on evidence demonstrating an inhibitory effect on GABA and glycine release induced by micromolar concentrations of Aβ (Mura et al., 2012; Zappettini et al., 2012), it can be speculated that perturbation of the inhibitory component of the excitatory/inhibitory network by Aβ may represent the neurochemical base underlying the appearance of psychotic symptoms (e.g., delusions, hallucinations, and misidentifications). In fact, the inhibitory component of the excitatory/inhibitory network plays a fundamental role in maintaining the excitatory/inhibitory functional balance in the brain, thus critically regulating cortical network function. In line with such hypothesis, mouse models recapitulating Aβ amyloidosis, generated by knock-in (KI) of a humanized Aβ sequence, exhibited behavioral changes associated with non-cognitive, emotional domains, before the onset of definitive cognitive deficits (Sakakibara et al., 2018; Latif-Hernandez et al., 2019). In Sakakibara's et al. (2018), assessments of the emotional domains showed that *App*-KI mice, harboring three familial AD-associated mutations (i.e., Swedish–NL–, Beyreuther/Iberian–F–, and Arctic–G–) (*App*^*NL*−*G*−*F*/*NL*−*G*−*F*^), developed progressive Aβ amyloidosis and exhibited anxiolytic-like behavior from 6 months of age, compared to wild-type mice. Instead, App-KI mice, carrying only the Swedish mutation (*App*^*NL*/*NL*^), displayed an anxiogenic-like behavior from 15 months of age. In the contextual fear conditioning task, while both *App*^*NL*/*NL*^ and *App*^*NL*−*G*−*F*/*NL*−*G*−*F*^ mice showed intact learning and memory up to 15–18 months of age, *App*^*NL*−*G*−*F*/*NL*−*G*^^−F^ mice had hyper-reactivity to painful stimuli. Such evidence indicates that anxiolytic-like behavior might be correlated with Aβ amyloidosis.

Noteworthy, although NPS are traditionally associated with frontotemporal dementia, the International Society to Advance Alzheimer's Research and Treatment (ISTAART) NPS Professional Interest Area developed diagnostic criteria to define the association between neuropsychiatric symptoms and other dementias, including AD, with the aim to define late-life appearance of sustained NPS as an at-risk condition for cognitive decline and dementia. Within this context, mild behavioral impairment (MBI) syndrome represents a diagnostic construct to identify patients with or without cognitive symptoms, prone to develop dementia, as well as a counterpart of mild cognitive impairment (MCI) and a transitional state between normal aging and dementia (Taragano et al., 2018). However, it is still unclear whether MBI represents a potentially reversible condition. Interestingly, Lussier et al. (2020) recently investigated the neuropathological correlates of MBI and found, as detailed below, an association between MBI and Aβ, but not tau or neurodegeneration, in cognitively intact elderly individuals. The authors investigated the association between the MBI Checklist (MBI-C) scores and AD imaging biomarkers (brain burden of Aβ, tau, and regional gray matter volume), in order to test whether MBI-C scores were correlated with early pathological stages of AD (Lussier et al., 2020). Higher MBI-C scores predicted higher Aβ PET labeling in the left frontal cortex, left posterior cingulate cortex, left caudate nucleus, and left thalamus, thus suggesting a correlation between MBI and amyloid pathology (Lussier et al., 2020). Notably, the areas with higher associations between MBI-C scores and Aβ PET uptake have been also reported to exhibit amyloidosis in the first phases of hierarchical amyloidosis in AD, specifically the neocortex, including frontal neocortex, followed by the striatum (Lussier et al., 2020). These results are consistent with evidence reporting that NPS are correlated with Aβ deposition in the frontal and cingulate cortices (Mori et al., 2014; Bensamoun et al., 2015) and subcortical amyloidosis (Hanseeuw et al., 2020). However, despite evidence showing that MBI represents an at-risk condition for dementia associated with Aβ deposition, it is still unknown which factors contribute to the progression from MBI to full-blown dementia and whether this progression is an extension of Aβ-driven detrimental effects.

## Concluding Remarks

Data from the literature, discussed in this mini review, highlight the key role of Aβ on synaptic activity and neurotransmission, in particular as endogenous modulator of neurotransmitter release from presynaptic terminals. However, our knowledge about the regulatory role of Aβ on synaptic activity and neurotransmission in the normal brain is extremely fragmentary and the application of exogenous Aβ has produced heterogeneous data on the topic, thus complicating the interpretation of the results discussed above. To date, it is unknown the mechanism by which endogenously released Aβ (comprising different isoforms and molecular conformations) modulates synaptic activity in normal and non-transgenic brain circuits (Abramov et al., 2009). Notably, such limitation hinders a clear comprehension of the biological mechanisms underlying the derangement from function to dysfunction and the switch of Aβ role from physiological to pathological.

Moreover, the overall scenario depicted in this paper raises a number of questions not yet fully resolved. First, a consideration comes from therapeutic endeavor targeting Aβ. Several thousands of patients have been treated with anti-Aβ drugs, ranging from strategies neutralizing Aβ with humanized monoclonal antibodies or promoting Aβ clearance, and these approaches have failed strong clinical goals. Based on the knowledge of a neuromodulatory role of Aβ, an antibody selectively binding and removing Aβ oligomers and fibrils might be more beneficial than one also directed to Aβ monomers. The failure of clinical trials testing Solanezumab, whose mechanism of action is peripheral sink and sequestration, may rely on its preference to bind to monomeric Aβ, since it recognizes a linear epitope in the center of Aβ and does not bind to larger Aβ aggregates (Willis et al., 2018). Some encouragement derives from aducanumab, a human monoclonal antibody selectively binding to Aβ fibrils and soluble oligomers, which in October 2019, after a reanalysis of the phase 3 studies, originally discounted after a futility analysis reporting no clinical advantage, showed some significant results (Schneider, 2020). However, it should be considered that the effects of aducanumab on cognitive decline were modest and severe side effects, such as cerebral edema, were observed, thus indicating that the risks may not be worth the benefits. Further considerations should be also done on the effect of a mobilization of Aβ from plaques, which appears detrimental and responsible for complications and severe side effects, such as amyloid-related imaging abnormalities (e.g., vasogenic edema and cerebral microhemorrhages) (Sperling et al., 2011; Mo et al., 2017).

Altogether, these questions suggest the importance of better analyzing the spectrum of Aβ effects to better frame the relevant time windows for intervention and to identify more appropriate targeting strategies.

## Author Contributions

FF, SG, and CL conceived the idea and wrote the manuscript. FF, SG, MR, and CL contributed in the critical discussion. All authors contributed to the article and approved the submitted version.

## Conflict of Interest

The authors declare that the research was conducted in the absence of any commercial or financial relationships that could be construed as a potential conflict of interest.
